# Radix Pseudostellaria polysaccharides alleviate sepsis-induced liver injury by modulating the gut microbiota via the TLR4/NF-κB pathway

**DOI:** 10.3389/fphar.2025.1658147

**Published:** 2025-09-24

**Authors:** Zhuolin Wang, Xiaohong Lin, Jianfeng Wu, Chanyuan Su, Yukun Luo, Guangwei Yu

**Affiliations:** ^1^ Department of Emergency, Fujian Medical University Union Hospital, Fuzhou, Fujian, China; ^2^ Fujian Key Laboratory of Vascular Aging, Fujian Medical University, Fuzhou, China; ^3^ Department of Cardiology, Fujian Medical University Union Hospital, Fuzhou, Fujian, China; ^4^ Department of Neurosurgery, Fujian Medical University Union Hospital, Fuzhou, Fujian, China

**Keywords:** gut-liver axis, multi-omics, Radix Pseudostellariae polysaccharides, sepsis, TLR4/NF-κB

## Abstract

**Background:**

Sepsis-induced liver injury (SLI) is a life-threatening complication with limited therapeutic options. Radix Pseudostellariae polysaccharides (RPPS), a component of traditional Chinese medicine, exert immunomodulatory, anti-inflammatory, and antioxidant properties. Herein, we investigated the therapeutic effects and mechanisms of RPPS on SLI.

**Methods:**

A murine sepsis model was established using cecal ligation and puncture. Mice were pretreated with RPPS or saline for 14 days. Subsequently, multi-omics integration—including metagenomics, proteomics, and network pharmacology—was employed to elucidate the mechanisms of RPPS. Liver injury was assessed via serum biomarkers, histopathology, and transmission electron microscopy, while intestinal barrier integrity was evaluated through histopathological analysis. Gut microbiota composition and functional pathways were examined using metagenomic sequencing. Furthermore, Kyoto Encyclopedia of Genes and Genomes enrichment analyses of gut microbiota, liver proteomics, and network pharmacology data were integrated to predict key target pathways, which were experimentally validated in mice.

**Results:**

RPPS pretreatment significantly improved survival, reduced liver injury markers, attenuated hepatic necrosis and inflammation, and restored intestinal barrier integrity. RPPS also modulated the gut microbiota by enriching beneficial taxa and suppressing pathogens. Multi-omics integration identified the toll-like receptor 4 (TLR4)/nuclear factor kappa-light-chain-enhancer of activated B cells (NF-κB) pathway as the core mechanism, and experimental validation confirmed that RPPS inhibited TLR4 membrane expression, MyD88/IKKα/β activation, NF-κB p65 phosphorylation, and nuclear translocation. In conclusion, RPPS alleviates SLI by protecting the intestinal barrier, modulating gut microbiota, and suppressing the TLR4/NF-κB signaling pathway.

**Conclusion:**

This study provides a scientific foundation for RPPS as a potential therapeutic candidate in sepsis treatment.

## Graphical Abstract

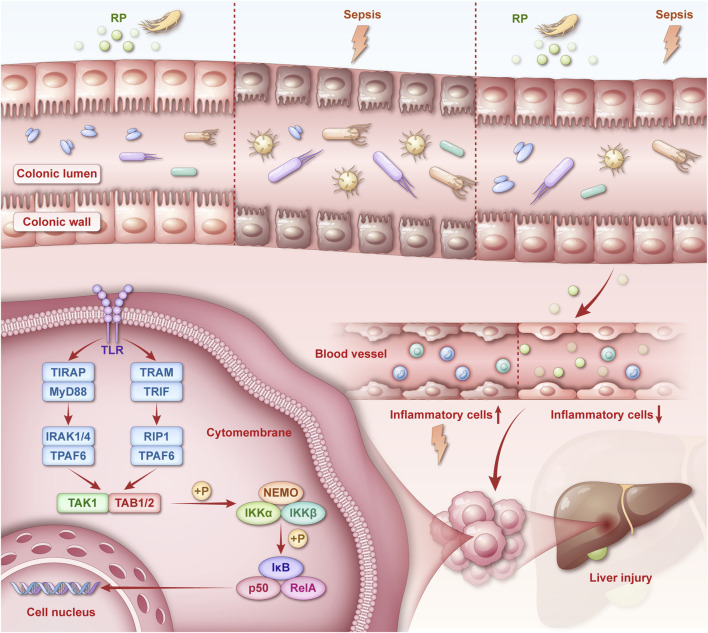

## 1 Introduction

Sepsis is an uncontrolled host response to infection that leads to life-threatening organ dysfunction (Singer et al., 2016). The pathophysiological mechanisms underlying sepsis are complex and involve dysregulated inflammation, metabolic reprogramming, and multi-organ crosstalk, particularly between the gut, liver, and immune system (Borges and Bento, 2024; Lelubre and Vincent, 2018). Sepsis-induced liver injury (SLI) is a life-threatening complication driven by hepatocyte apoptosis, disrupted hepatic homeostasis, and systemic inflammation. SLI can disrupt hepatocyte function, trigger apoptosis, and damage liver tissue (Savio et al., 2017; Wang et al., 2014). The gut–liver axis plays a central role in SLI pathogenesis: sepsis disrupts the gut microbiota, impairs the intestinal barrier, and promotes bacterial migration with endotoxin entry into portal circulation, driving hepatic immune activation and intensifying liver injury (Moura and Siqueira AI de, 2025; Zhang et al., 2022). Restoring the gut microbiota balance significantly attenuates SLI, highlighting the therapeutic potential of microbiota modulation (Gong et al., 2019).

Traditional Chinese medicine (TCM) polysaccharides are promising therapeutic agents that target the gut–liver axis and have anti-inflammatory and probiotic effects, similar to those of fecal microbiota transplantation (Yuan et al., 2025). TCM polysaccharides inhibit inflammation, accelerate bacterial clearance, and enhance cell survival (Bian et al., 2021; Gao et al., 2021). Radix Pseudostellariae (RP), a ginseng herb rich in polysaccharides, has strong immunomodulatory and anti-inflammatory properties. Emerging evidence suggests that RP polysaccharides (RPPS) may regulate the intestinal microenvironment and mitigate organ damage (Zhou et al., 2021). However, the underlying mechanisms by which they mitigate, particularly their influence on signaling pathways, remain poorly understood.

In this study, we sought to explore RPPS’ hepatoprotective effects against SLI using a murine sepsis model and a multi-omics approach, including metagenomic sequencing to characterize microbiota alterations, liver proteomics to identify perturbed pathways, and network pharmacology to pinpoint RPPS targets. We hypothesized that RPPS would alleviate SLI by restoring gut microbiota homeostasis, reinforcing intestinal barrier function, and suppressing Toll-like receptor 4 (TLR4)/nuclear factor kappa-light-chain-enhancer of activated B cells (NF-κB)-driven hepatic inflammation. This study determined the molecular basis for the therapeutic effect of RPPS and provided a paradigm for integrating TCM with systems biology to combat sepsis-induced organ dysfunction.

## 2 Materials and methods

### 2.1 Untargeted metabolomics by liquid chromatography-mass spectrometry

Liquid chromatography separation was performed using a Vanquish Horizon UHPLC system (Thermo Fisher Scientific, United States) equipped with an ACQUITY UPLC HSS T3 column (dimensions: 100 × 2.1 mm, particle size: 1.8 μm; sourced from Waters, Milford, MA, United States), maintained at a temperature of 40 °C. The mobile phase, comprising aqueous 0.1% formic acid (solvent A) and acetonitrile (solvent B), was delivered at a steady flow rate of 0.4 mL/min. The acquired data were processed using Progenesis QI software.

### 2.2 Network pharmacology analysis

RP components were acquired from the TCM Systems Pharmacology Database and Analysis Platform (TCMSP, https://old.tcmsp-e.com/tcmsp.ph, Version 2.3). Components were screened based on key pharmacokinetic properties (drug-likeness score >0.18; oral bioavailability >30%) to identify potential bioactive constituents (Ru et al., 2014). The active components of TCM were screened using SWISSADME (http://www.swissadme.ch/). Components with at least two “Yes” responses among the five drug-likeness criteria (Veber, Egan, Lipinski, Ghose, and Muegge) and a Pharmacokinetics (drug disposition) index of gastrointestinal absorption classified as “high” were selected for additional examination. Sepsis-related targets were retrieved from genomic (GenBank), therapeutic (Therapeutic Target Database), disease-associated (DisGeNET), and genetic disorder (Online Mendelian Inheritance in Man) databases.Targets from TCMSP/Swiss Target Prediction were deduplicated for network construction. Venn analysis identified shared targets (Bardou et al., 2014). Intersection targets were imported into the STRING 11.5 database (https://www.string-db.org/), and a protein-protein interaction (PPI) network was constructed (Szklarczyk et al., 2019). Gene Ontology (GO) analyses and Kyoto Encyclopedia of Genes Genomes (KEGG) pathway enrichment were conducted on overlapping targets using the clusterProfiler package (R package version 4.6.2, Vienna, Austria) (Zhou et al., 2019).

### 2.3 Animal experiment

**FIGURE 1:**
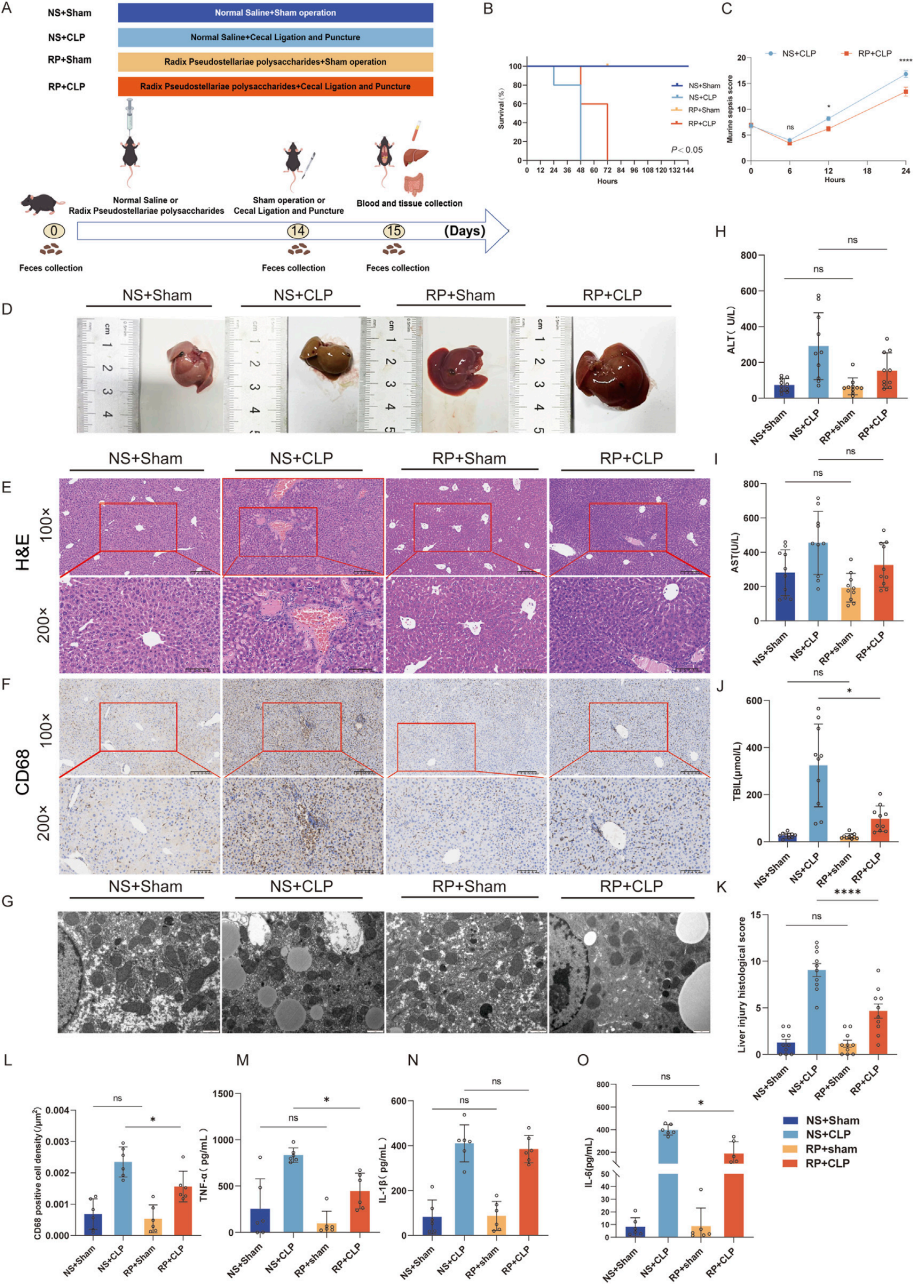
Radix Pseudostellariae polysaccharides (RPPS) attenuate CLP-induced liver inflammation and injury in mice. **(A)** The experimental design and procedures for RPPS administration. **(B)** Survival rate. **(C)** MSS in the NS + CLP and RP + CLP groups. **(D)** Liver morphology. **(E)** Histological representation of liver sections using hematoxylin and eosin **(H,E)** staining (×100 and ×200 magnification, n = 10). **(F)** Immunohistochemical staining of CD68 (×100 and ×200 magnification, n = 6). **(G)** Representative transmission electron microscopy images of hepatocytes. Bar = 1 μm (n = 6–8). **(H–J)** Alanine aminotransferase (ALT), aspartate aminotransferase (AST), and total bilirubin (TBIL) levels. **(K)** Histopathological score from H&E sections. **(L)** The rate of CD68-positive cell density in the liver. **(M–O)** Levels of TNF-α, IL-1β, and IL-6 in liver tissue. Data are presented as mean ± standard error of mean. A one-way analysis of variance with Tukey’s analysis was used for multiple comparisons. Significance levels are represented as: *p < 0.05, **p < 0.01, ***p < 0.001, ****p < 0.0001, with “ns” indicating “not significant.”

C57BL/6J mice of Male Specific Pathogen Free grade were obtained from Yaokang Biotechnology Co., Ltd. (Guangdong, China). The body weight of the mice was 26.87 ± 1.80 g at 12 weeks of age. Following IACUC approval (FJMU 2024-Y-1895), all animals were acclimated at 23 °C under a 12-h light-dark cycle. Cecal ligation and puncture were performed using standard protocols. (Rittirsch et al., 2009). The sham groups underwent laparotomy without cecal intervention. The mice were stratified into four groups (n = 10): NS (Normal Saline)+Sham, NS + CLP, RP + Sham, and RP + CLP. The RP groups received 14-day RPPS pretreatment (200 mg/kg/day via gavage), whereas the NS groups received the same volume of NS (Figure 1A). Fecal samples were categorized as pre-/post-NS/RP. All mice were anesthetized using isoflurane. CLP groups underwent a 1.5-cm midline incision, ligation of the distal one-third of the cecum, and two punctures with a 14-G needle, followed by gentle fecal extrusion. The incision was closed in layers, and 1 mL of normal saline was administered subcutaneously for fluid resuscitation. Independent survival cohorts (n = 5) were tracked for 144 h post-surgery. Postoperative protocols included thermoregulation (36 °C–38 °C) and *ad libitum* access.

### 2.4 Biochemical parameters

Twenty-four hours post-CLP or sham operation, mice were anesthetized via intraperitoneal administration of sodium pentobarbital (2.5%, 2.5 mL/kg) and euthanized by cervical dislocation. Blood samples were subsequently collected for hepatic function analysis. Serum concentrations of total bilirubin (TBIL), aspartate aminotransferase (AST), and alanine aminotransferase (ALT) were quantified using an AU5800 Clinical Chemistry Analyzer (Beckman Coulter, Brea, CA, United States) at the Fujian Medical University Union Hospital in China.

### 2.5 Histologic analysis

The mice were euthanized 24 h post-CLP or sham surgery for colon and liver tissue collection. Following fixation in 4% neutral buffered formalin, tissues underwent paraffin embedding, sectioning at 5-μm thickness, and hematoxylin-eosin (H&E) staining (Li et al., 2022). H&E-stained tissue sections were subjected to histopathological scoring of colon and liver tissues following established protocols from prior studies (detailed in supplementary material) (Guo et al., 2021; Liang et al., 2022). Following deparaffinization and rehydration, tissue sections underwent Periodic acid-Schiff (PAS) staining to visualize goblet cells. We quantified results by enumerating all PAS-positive cells in structurally intact colon crypts (Shin et al., 2021).

### 2.6 Cytokine levels

Hepatic concentrations of interleukin (IL)-1β, IL-6, and tumor necrosis factor (TNF)-α were quantified using species-specific ELISA kits (Thermo Fisher Scientific; catalog #88-7013, 88-7064, 88-7324) following the manufacturer’s protocols.

### 2.7 Immunohistochemical analysis

CD68, TNF-α, and IL-1β expression levels were measured with an immunohistochemistry (IHC) kit (PK10006, Proteintech, United States) for rabbit/mouse primary antibodies (Fang et al., 2022). Deparaffinization, rehydration, and antigen retrieval were carried out using trypsin incubation (003009, Thermo Fisher Scientific) for 30 min or heat-mediated retrieval for 20 min. Subsequently, slides were blocked with 10% goat serum (SL038, Solarbio, China) at room temperature for 45 min. Primary antibodies against CD68: 1:200, 28058-1-AP; TNF-α: 1:200, 26405-1-AP; IL-1β: 1:200, 26048-1-AP, all sourced from Proteintech, were incubated at 4 °C for 16 h. Endogenous peroxidase activity was blocked by 20-min incubation in 3% hydrogen peroxide. Secondary antibody incubation (30 min) was performed the next day. Finally, DAB staining and hematoxylin counterstaining were performed before sealing the slides with DPX mounting medium (06522, Sigma, St. Louis, MO, United States) for microscopic examination.

### 2.8 Immunofluorescence assay

Colon and liver tissue sections underwent immunofluorescence staining according to established protocols (Pan et al., 2024). The primary antibodies used were anti-Occludin (1:1000, 27260-1-AP; Proteintech), anti-TLR4 (1:200, 19811, Proteintech) and anti-phospho-NF-κB p65 (1:800, #3033, CST, Danvers, MA, United States). The secondary antibodies employed were Alexa Fluor 488 conjugated goat anti-rabbit IgG (1:400, GB25303, Servicebio, Wuhan, China) and Cy3-conjugated goat anti-rabbit IgG (1:300, GB21303, Servicebio). Following standard deparaffinization and antigen retrieval protocols, liver sections incubated with primary antibodies at 4 °C for 16 h, followed by incubation with secondary antibodies at room temperature for 50 min under light-protected conditions. Post-PBS washing cycles, nuclear counterstaining was performed using DAPI (G1012, Servicebio). Fluorescent signals were documented through a fluorescence microscope (ECLIPSE C1, Nikon, Tokyo, Japan), with three randomly selected microscopic fields per specimen subjected to quantitative analysis using ImageJ software (NIH, Bethesda, MD, United States).

### 2.9 Protein extraction and Western blot analysis

Briefly, hepatic protein extraction was performed with RIPA lysis buffer (G2002, Servicebio) via centrifugation at 12,000 × g for 5 min (4 °C). Appropriate amounts of SDS loading buffer (G2075, Servicebio) were added and boiled. Following electrophoretic separation, proteins were electro-transferred onto polyvinylidene fluoride membranes (Merck Millipore, United States). Membranes underwent 30-min room temperature blocking with Rapid Blocking Buffer (Servicebio, #G2052) prior to overnight incubation with primary antibodies at 4 °C, including anti-NF-κB (1:1,000, #8242, Cell Signaling, Danvers, MA, United States), antiphospho-NF-κB (1:1000, #3033, Cell Signaling), anti-MyD88 (1:1000, #4283, Cell Signaling), anti-βActin (1:1000, #4970, Cell Signaling), and anti-IKKα/β (1:2000, PT0435R, Immunoway, San Jose, China) overnight at 4 °C. Then the membranes were incubated with the respective secondary antibodies, including anti-rabbit IgG (1:1000, GB23303, Servicebio) for 40 min at room temperature. Bands were detected using enhanced chemiluminescent liquid (ECL, G2020, Servicebio) and images acquisition were conducted with the ChemiDoc MP imaging system (Bio-Rad Laboratories, United States). Blotting analysis was conducted using ImageJ software.

### 2.10 Gut microbiota analysis

Gut microbiota profiling was conducted via shotgun metagenomic sequencing. Fecal DNA samples underwent mechanical fragmentation (M220, Covaris, Woburnn, MA, United States) to 350-bp fragments for paired-end library preparation with NEXTflex Rapid DNA-Seq kits (Bioo Scientific, Austin, TX, United States). Sequencing was executed on Illumina NovaSeq X Plus (Illumina, San Diego, CA, United States) using NovaSeq X Series 25B reagents per manufacturer’s protocols, encompassing DNA ligation, purification, and amplification. Raw reads were quality-filtered through fastp (adapter trimming; read length≥50 bp; Q ≥ 20, version 0.20.0) (Chen et al., 2018). High-quality sequences were co-assembled via MEGAHIT (contig length≥300 bp, v1.1.2) (Li et al., 2015). Metagene-predicted Open reading frames (length≥100 bp; Prodigal v2.6.3) were functionally annotated against KEGG database (v94.2). A non-redundant gene catalog was generated using CD-HIT v4.7 (90% identity/coverage), with gene abundance quantified via SOAPaligner v2.21 (95% identity) (Li et al., 2008). Taxonomic assignment employed DIAMOND (v2.0.13) against NCBI NR (e-value≤1e-5) (Buchfink et al., 2015). Multi-level differential analysis (taxonomic/functional/gene) was performed using Kruskal–Wallis tests based on annotated profiles. The analyses related to bioinformatics were executed via the Majorbio Cloud Platform (www.majorbio.com).

### 2.11 Proteomics

Murine hepatic tissues were processed by immersion in lysis buffer at a 1:100 weight-to-volume ratio for 2 h, followed by ultrasonication and centrifugation at 12,000×g. Subsequently, supernatants were treated with 10 mM DTT and 5 mM IAA for 30 min, and tryptic digestion was carried out for 16 h. Peptide separation was achieved via Q-Exactive Plus MS (Thermo Fisher) with a mobile phase comprising 0.1% formic acid (A) and acetonitrile (B), delivered at 0.4 μL/min through a 90-min linear gradient (4%–35% B). MS/MS spectra were matched against the UniProt database using MaxQuant (v1.6.17) for label-free quantification. Functional annotation was implemented using KEGG (v94.2). Bioinformatics analyses were conducted using the Majorbio Cloud Platform.

### 2.12 Transmission electron microscopy

Samples of liver tissue, sized between 1 and 3 mm^3^, were collected and immediately fixed for transmission electron microscopy (Gao et al., 2021). Following post-fixation with 1% osmic acid, the tissue blocks were dehydrated using ethanol and acetone gradients, embedded in Epon-812 resin, and polymerized overnight. The samples were cut into 70-nm ultrathin sections and, then, examined and photographed using a JEM1400 transmission electron microscope (JEOL, Tokyo, Japan).

### 2.13 Molecular docking

The three-dimensional (3D) structure of the core target protein TLR4 (in PDB format) was retrieved from the Protein Data Bank (PDB; http://www.rcsb.org/pdb/) (Berman et al., 2000). Molecular structures (in SDF format) of key active compounds—1-monolinolein, acacetin, β-sitosterol, linarin, luteolin, schottenol, upraene, and taraxerol—were acquired from the PubChem database (https://pubchem.ncbi.nlm.nih.gov/) (Kim et al., 2023). Molecular docking between the core target protein and active compounds was performed using CB-Dock2 (https://cadd.labshare.cn/cb-dock2/php/index.php) (Liu et al., 2022).

### 2.14 Statistical analysis

GraphPad Prism (Version 9.0) was used for data analysis, and the results are shown as mean values with the standard error of the mean. The Shapiro-Wilk tests were used to assess the normality of data distribution. For normally distributed data, the one-way analysis of variance was used for multigroup comparisons. For non-normally-distributed data, the Mann–Whitney *U*-test was used for comparisons of two groups, while the Kruskal–Wallis test was used for multigroup comparisons. Two-tailed *p* values of <0.05 were considered statistically significant.

## 3 Results

### 3.1 RPPS alleviates SLI and inhibits inflammation in the liver of mice

Results of qualitative analysis of RPPS are summarized in Supplementary Figure S1. The survival rates of the mice are shown in Figure 1B. The survival rate of mice in the NS + CLP group was lower than that of mice in the RP + CLP group (*p* < 0.05). The Murine Sepsis Score (MSS) (Shrum et al., 2014) calculated in two groups of septic mice showed that the RP + CLP group scored significantly lower than the NS + CLP group (*p* < 0.0001) (Figure 1C).

Liver morphology is presented in Figure 1D. H&E staining revealed that CLP induced marked congestion, inflammatory cell infiltration, necrosis, and degeneration in the liver. RPPS pretreatment attenuated the pathological changes in the liver (Figure 1E). Compared with the NS + CLP group, the RP + CLP group had less histological damage (*p* < 0.0001) (Figure 1K) and less macrophage infiltration (Figures 1F,L).

Following CLP, transmission electron microscopy of liver tissues revealed hepatocyte mitochondrial matrix degradation, partial disruption of cristae structure, moderate mitochondrial edema, and mild endoplasmic reticulum dilation and degranulation, whereas liver tissues in the Sham group displayed predominantly normal characteristics (Figure 1G). The mitochondrial changes were less marked in the RP + CLP group compared to those in the NS + CLP group.

The ALT, AST, and TBIL levels increased after CLP and were significantly higher in the NS + CLP group compared to those in the RS + CLP group (Figures 1H–J). Moreover, the TNF-α and IL-6 levels were significantly higher in the NS + CLP compared to those in the RP + CLP groups. These results demonstrate that RPPS has a protective effect on the liver during sepsis.

### 3.2 RPPS attenuates CLP-induced intestinal barrier impairment

**FIGURE 2:**
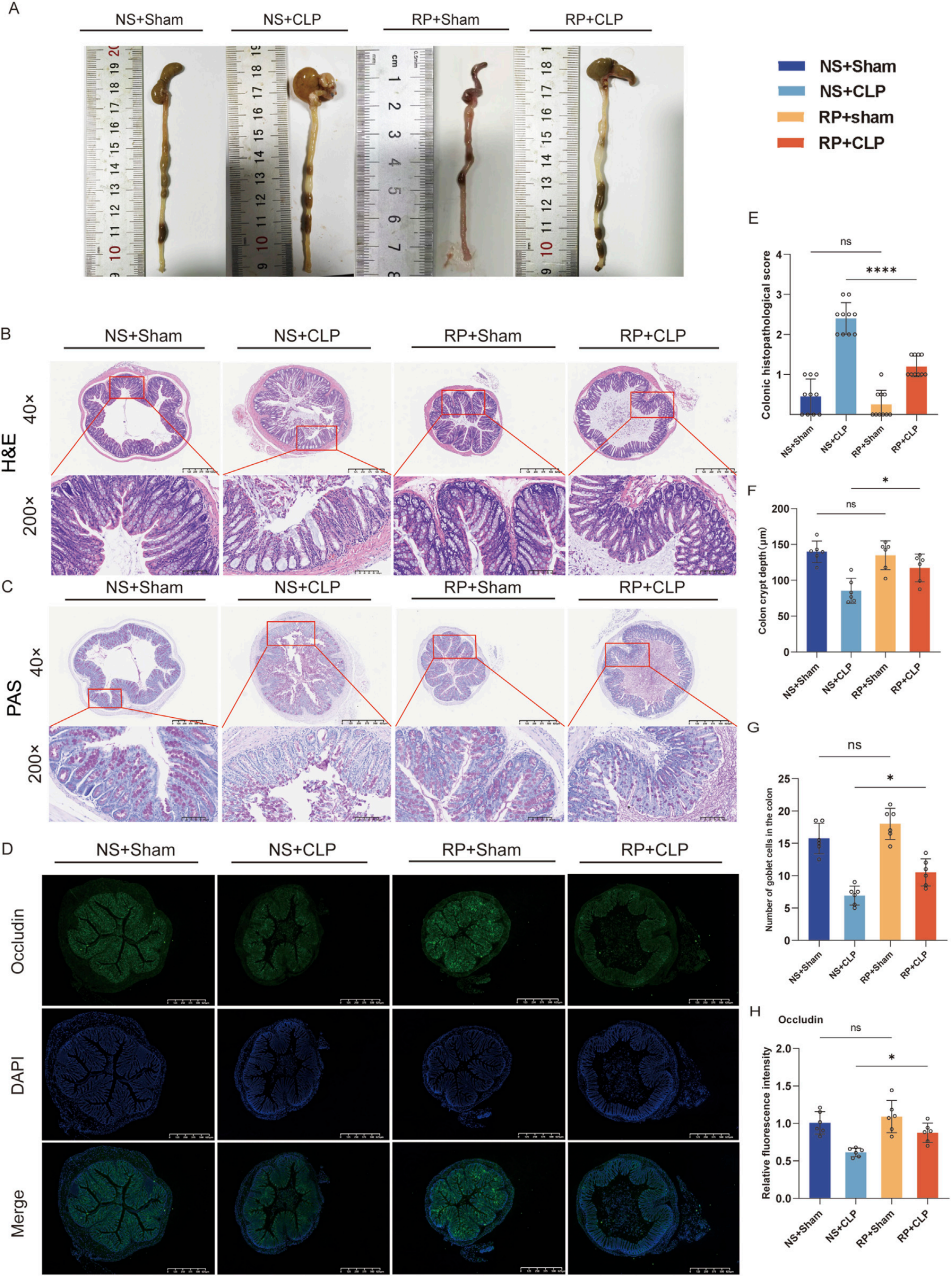
Radix Pseudostellariae polysaccharides (RPPS) restore intestinal morphology and intestinal barrier function in cecal ligation and puncture (CLP) sepsis-induced mice. **(A)** Colon morphology. **(B,C)** Histological representation of colonic sections using hematoxylin and eosin (H&E) and Periodic acid-Schiff (PAS) staining. (×40 and ×200 magnification, n = 6–10) **(D)** Immunofluorescence staining of occludin in colonic sections. **(E)** Histopathological scoring from H&E sections. **(F)** Colon crypt depth of each group from H&E sections (n = 6). **(G)** The number of goblet cells in the colon from PAS sections (n = 6). **(H)** The occludin expression in colon tissues; the scale bar represents 625 μm (n = 6). Data are presented as mean ± standard error of mean. A one-way analysis of variance with Tukey’s analysis was used for multiple comparisons. Significance levels are represented as: *p < 0.05, **p < 0.01, ***p < 0.001, ****p < 0.0001, with “ns” indicating “not significant.”

Colon pictures showed that intestinal adhesion in NS + CLP group was more serious than that in other groups (Figure 2A). H&E and PAS staining showed no significant differences in the colonic tracts of mice in the NS + Sham and the RP + Sham groups (Figures 2B,C,H) or in the crypt depth and goblet cells between these groups (Figures 2F,G).

However, the mice in the NS + Sham and the RP + Sham groups exhibited significantly lower histopathological scores for colonic injury compared to those in the NS + CLP and RP + CLP groups (Figure 2E). Furthermore, the crypt depth in the RP + CLP group was significantly greater than that in the NS + CLP group (*p* < 0.05; Figure 2F), while the number of goblet cells was significantly lower in the NS + CLP group compared to the corresponding in the RP + CLP group (*p* < 0.05; Figure 2G). Additionally, immunofluorescence staining revealed that occludin expression in the NS + CLP group significantly decreased compared with that in the RP + CLP group (*p* < 0.01; Figures 2D,H).

### 3.3 RPPS regulates CLP-induced gut microbiota dysbiosis in septic mice

**FIGURE 3:**
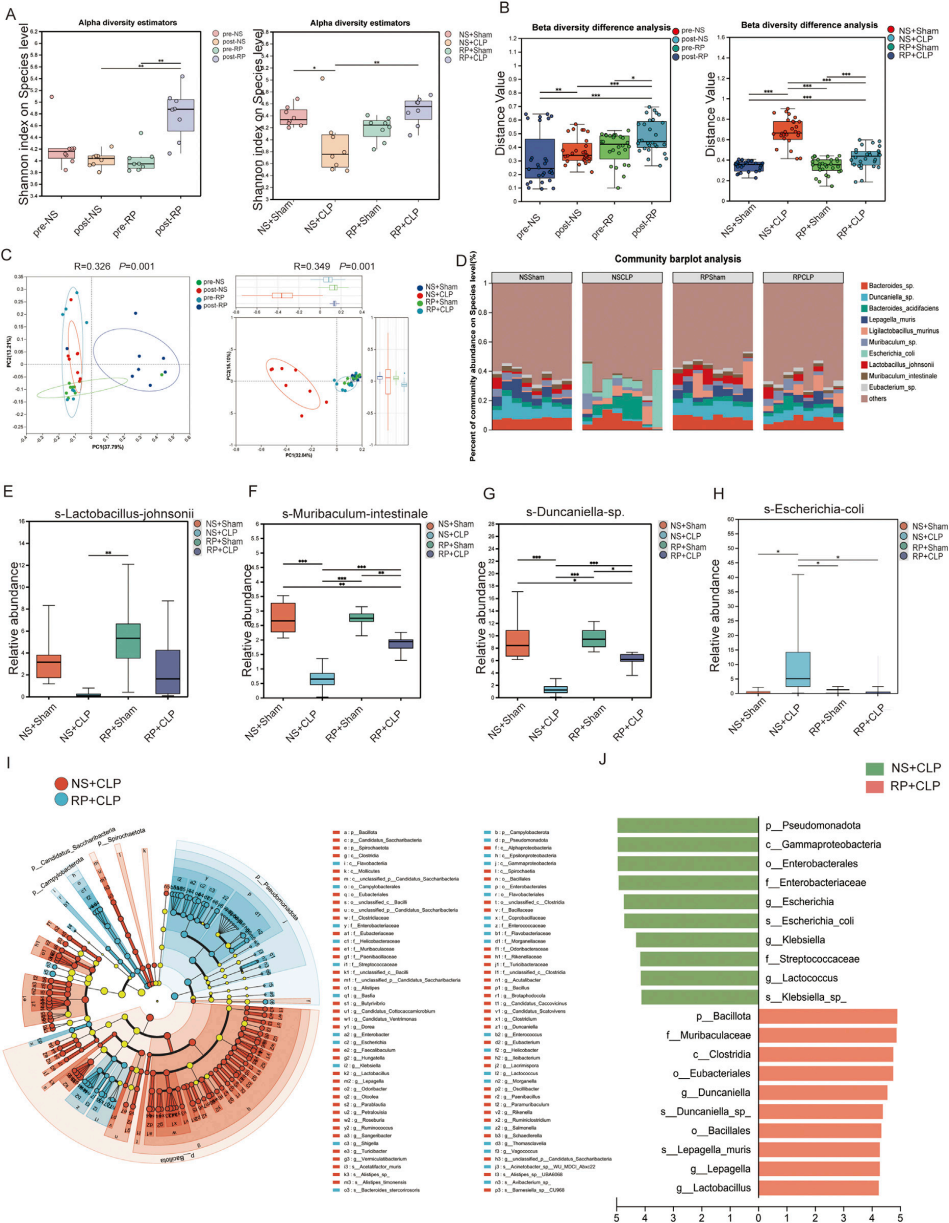
Radix Pseudostellariae polysaccharides (RPPS) modulate the composition of gut microbiota. **(A)** Alpha diversity of gut microbiota. **(B)** Beta diversity difference analysis. **(C)** Principal Coordinate Analysis (PCoA) analysis at the species level. **(D)** Bar plots of the relative abundance of intestinal microbiota at the species level. **(E–H)** The relative abundance of *Lactobacillus johnsonii, Muribaculum intestinale*, *Duncaniella* spp. increased, and that of *Escherichia coli* decreased across different groups. **(I,J)** Linear discriminant analysis Effect Size (LEfSe) Hierarchical Clustering Tree Plot and the histogram of linear discriminant analysis (LDA) between the NS + CLP and RP + CLP groups. Data are expressed as the mean ± SD. An LDA score of >4.0 was considered statistically significant. Significance levels are represented as: *p < 0.05, **p < 0.01, ***p < 0.001, ****p < 0.0001, with “ns” indicating “not significant.” Kruskal–Wallis rank-sum test (Dunn’s test) in **(A)**, Wilcoxon rank-sum test in **(B)**, Kruskal–Wallis H-test at a species level in **(E–H)**, Spearman’s rank-correlation coefficients were calculated for correlation analysis, *p* < 0.05; n = 6–8.

Metagenomic analysis indicated that the α-diversity indices of gut microbiota, measured by the Shannon index, were significantly higher in the post-RP group compared to the post-NS and pre-RP groups (Figure 3A). Moreover, the Shannon index was lower in the NS + CLP compared to that in the NS + Sham group; the RP + CLP group showed a higher Shannon index than the NS + CLP group (Figure 3A). The Chao and ACE index results are provided in Supplementary Figure S4A. These results demonstrate that RPPS pretreatment counteracted sepsis-related alpha diversity loss following CLP.

To assess inter-group microbial heterogeneity, β-diversity analysis revealed distinct clustering patterns across cohorts. Notably, the post-RP group exhibited unique microbial profiles compared to the pre-NS, post-NS, and pre-RP groups, while RP + CLP specimens demonstrated greater phylogenetic similarity to the NS + Sham group than to the NS + CLP group (Figure 3B). Principal co-ordinates analysis plots confirmed pronounced compositional segregation in post-RP specimens relative to other groups (Figure 3C). Importantly, NS + CLP microbiomes displayed marked divergence from all groups, indicating that RPPS pretreatment effectively ameliorated sepsis-induced dysbiosis. Species-resolved microbiota profiling (Figure 3D) identified significant dysbiosis in the NS + CLP cohort, characterized by depleted abundances of *Lactobacillus*, *Muribaculum intestinale*, and *Duncaniella* species, contrasted with enriched *Escherichia coli* colonization compared to the other three groups. The RP + Sham group showed a higher relative abundance of *Ligilobacillus* murinus compared to the NS + Sham group. (Figures 3E–H). The composition of gut microbes in the RP + CLP group more closely resembled that of the RP + Sham and NS + Sham groups than that of the NS + CLP group. Moreover, *Lactobacillus* abundance was significantly higher in the post-RP group compared to the post-NS group (Supplementary Figure S4B). Additionally, linear discriminant analysis effect size from phylum to species level revealed distinct bacterial lineages between the NS + CLP and RP + CLP groups. Notably, the NS + CLP group exhibited significantly higher relative abundances of *g-Lactococcus*, *f-Streptococcaceae*, *g-Klebsiella*, *s-Escherichia coli*, *g-Escherichia*, *f-Enterobacteriaceae*, *o-Enterobacterales*, *c-Gammaproteobacteria*, *p-Pseudomonadota*, *g-Lactobacillus*, *g-Lepagella*, *s-Lepagella muris*, *o-Bacillales*, *s-Duncaniella* sp., *g-Duncaniella*, *o-Eubacteriales*, *c-Clostridia*, *f-Muribaculaceae*, and *p-Bacillota* (Figures 3I,J).

**FIGURE 4:**
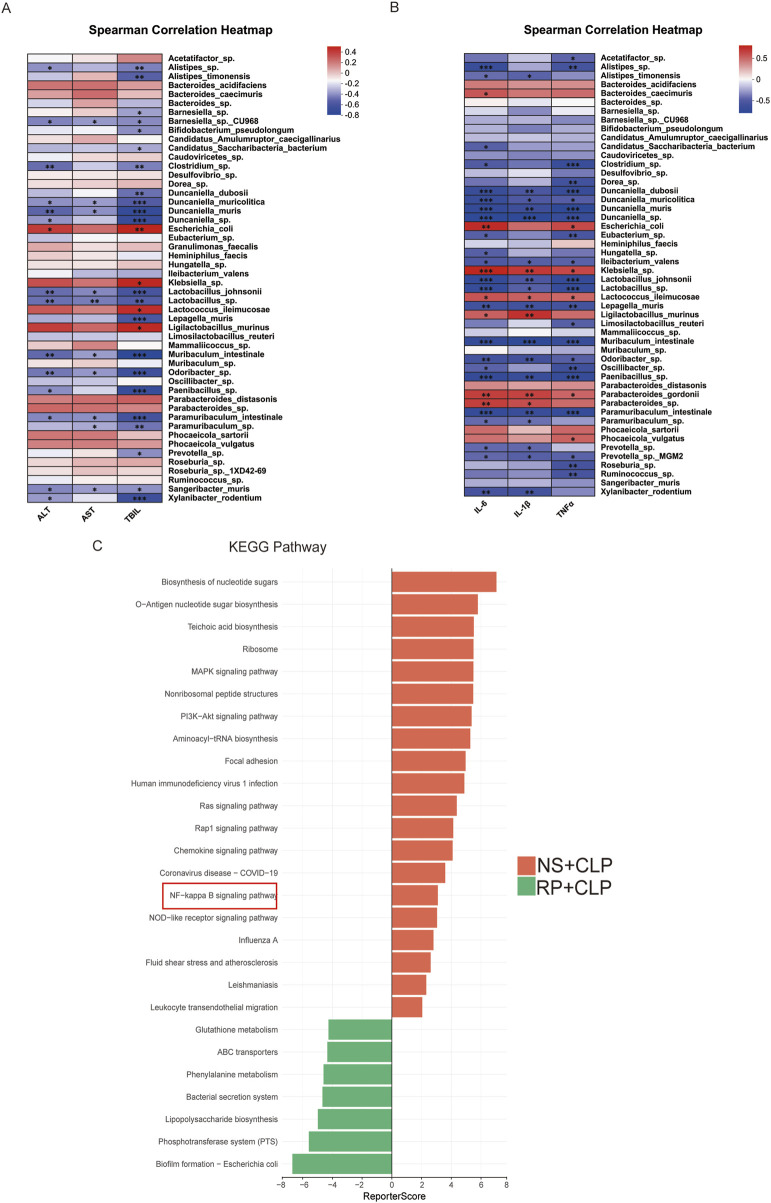
Gut microbiota is correlated with liver function and hepatic inflammatory factors. **(A)** Correlation heatmap of intestinal microbiota and hepatic inflammatory cytokines. **(B)** Correlation heatmap of intestinal microbiota and liver function. **(C)** KEGG enrichment assay between the NS + CLP and RP + CLP groups.

Correlation analysis revealed statistically significant associations between hepatic injury biomarkers (ALT, AST, TBIL) and specific gut microbes. Positive correlations were observed with *Escherichia coli* and *Klebsiella*, whereas inverse correlations were noted with commensal taxa, such as *Bifidobacterium*, *Lactobacillus*, and *Muribaculum intestinale* (Figure 4A). Similarly, analysis of gut microbiota composition in relation to hepatic inflammatory markers identified positive associations of *Klebsiella* and *Escherichia coli* with TNF-α, IL-6, and IL-1β, alongside inverse relationships with commensal taxa including *Bifidobacterium*, *Lactobacillus*, and *Muribaculum intestinale* (Figure 4B). Furthermore, KEGG pathway analysis demonstrated significant enrichment of the NF-κB pathway in the NS + CLP group compared to the RP + CLP group (Figure 4C). Collectively, these findings suggest that RPPS may mitigate SLI via mechanisms involving the gut–liver axis and suppression of NF-κB signaling.

### 3.4 Proteomics indicates that the NF-κB signaling pathway is critical in the phenotype of sepsis following treatment with RP

**FIGURE 5:**
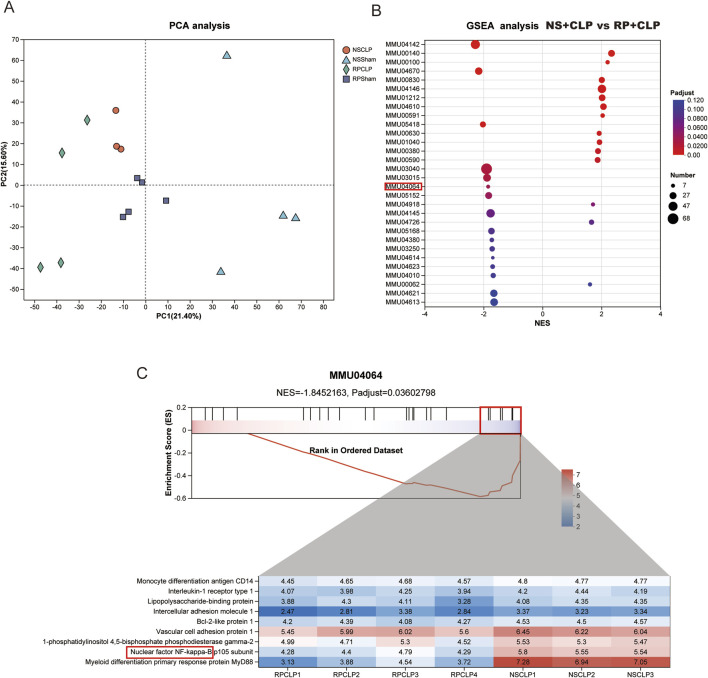
Proteomics reveals the critical role of NF-κB signaling pathway in the phenotype of sepsis-induced liver injury (SLI) following Radix Pseudostellariae polysaccharides (RPPS) treatment. **(A)** Principal component analysis (PCA) of liver proteomes. **(B)** Gene set enrichment analysis (GSEA) comparing RP + CLP and NS + CLP groups, highlighting significant enrichment of the NF-κB signaling pathway in the RP + CLP group. **(C)** Detailed GSEA results for the MMU04064 gene set showing differential expression of NF-κB pathway proteins between the RP + CLP and NS + CLP groups.

To explore the mechanism of action of RPPS, we conducted a comprehensive proteomic analysis of liver tissues. The principal component analysis (PCA) results demonstrated clear separation and clustering of samples from different groups, highlighting the differences in liver protein expression profiles (Figure 5A). The gene set enrichment analysis comparing the RP + CLP and NS + CLP groups revealed a notable enrichment of the NF-κB pathway in the NS + CLP group, characterized by a normalized enrichment score (NES) of −1.8452163 and an adjusted *p* value (*p* adjust) of 0.03602798 (Figures 5B,C). Analysis of the NF-κB signaling pathway proteins within the MMU04064 gene set revealed distinct expression patterns between the RP + CLP and NS + CLP groups, as visualized by the heatmap. Notably, key proteins in the NF-κB pathway, including Myd88, were downregulated in the RP + CLP group in contrast to the NS + CLP group (Figure 5C).

### 3.5 Network pharmacology reveals multiple signaling pathways involved in RPPS’s impact on sepsis

**FIGURE 6:**
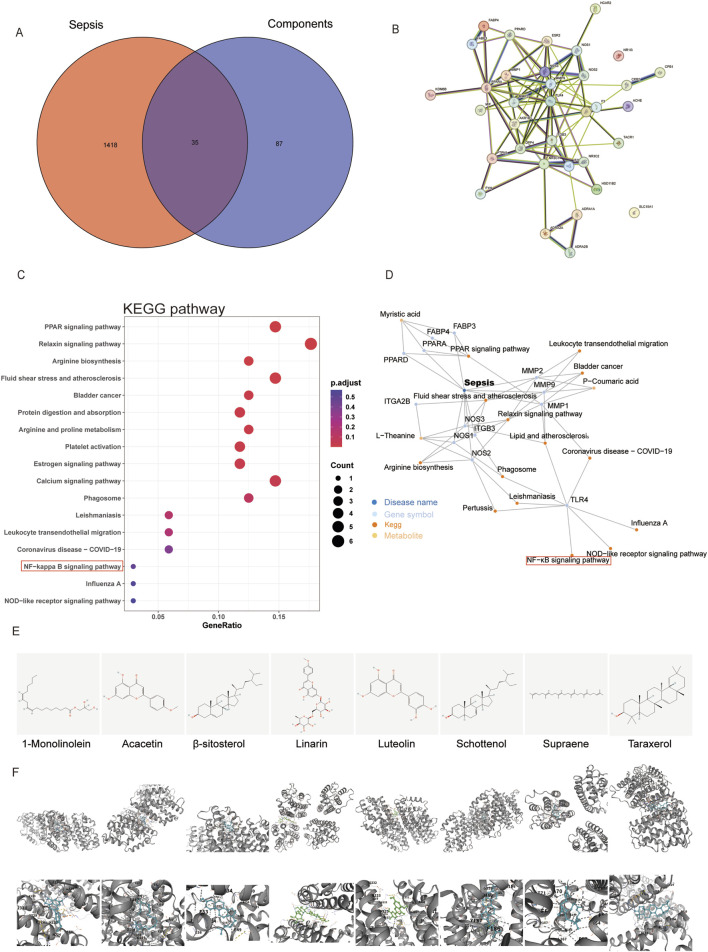
Prediction of targets of Radix Pseudostellariae polysaccharides (RPPS) treatment in sepsis-induced liver injury (SLI). **(A)** The common target of RPPS in the treatment of SLI. **(B)** protein–protein interaction (PPI) network of common targets. **(C)** KEGG enrichment analysis of core targets. **(D)** Potentially active ingredients that intersect with sepsis targets. **(E)** The chemical structures of the screened small molecules. **(F)** Molecular docking between the TLR4 and screened small molecules.

In the TCMSP database, we screened eight potentially active RPPS ingredients. Subsequently, the drug targets were categorized and intersected with sepsis targets. A total of 35 overlapping targets were identified, indicating potential RPPS targets for sepsis alleviation (Figure 6A). The intersection target data were imported into the STRING11.5 database and a PPI network was constructed specifying “human/mouse” as the study species (Figure 6B). Key targets for disease treatment were identified based on an average value exceeding the Degree value, along with their associated targets and active ingredients. The KEGG pathway analysis of the overlapping targets revealed the following top 17 enriched pathways: hsa04926, Relaxin signaling pathway; hsa03320, PPAR signaling pathway; hsa00220, Arginine biosynthesis; hsa05418, Fluid shear stress and atherosclerosis; hsa05219, Bladder cancer; hsa04974, Protein digestion and absorption; hsa00330, Arginine and proline metabolism; hsa04611, Platelet activation; hsa04915, Estrogen signaling pathway; hsa04020, hsa05140, Leishmaniasis; Calcium signaling pathway; hsa04145, Phagosome; hsa04670, Leukocyte transendothelial migration; hsa04064, NF-κB signaling pathway; hsa05171, Coronavirus disease - COVID-19; hsa05164, Influenza A; hsa04621, NOD-like receptor pathway (Figure 6C). These pathways are mainly linked to immune responses, inflammation, oxidative stress, and metabolic regulation. A network diagram was drawn based on the relationships between TCM, active ingredients, targets, and pathways (Figure 6D). Eight key active ingredients (Figure 6E) were subjected to molecular docking with the core targets TLR4. The binding affinity between the active ingredients and the targets was assessed based on the binding energy values (a binding energy of < -4.25 kcal/mol indicates a certain level of binding activity; <-5.0 kcal/mol indicates a higher level of binding activity; and <-7.0 kcal/mol indicates very strong binding activity). There were eight best binding results (Figure 6F).

### 3.6 RPPS inhibits the NF-κB pathway and reduces liver inflammation in mice with sepsis

**FIGURE 7:**
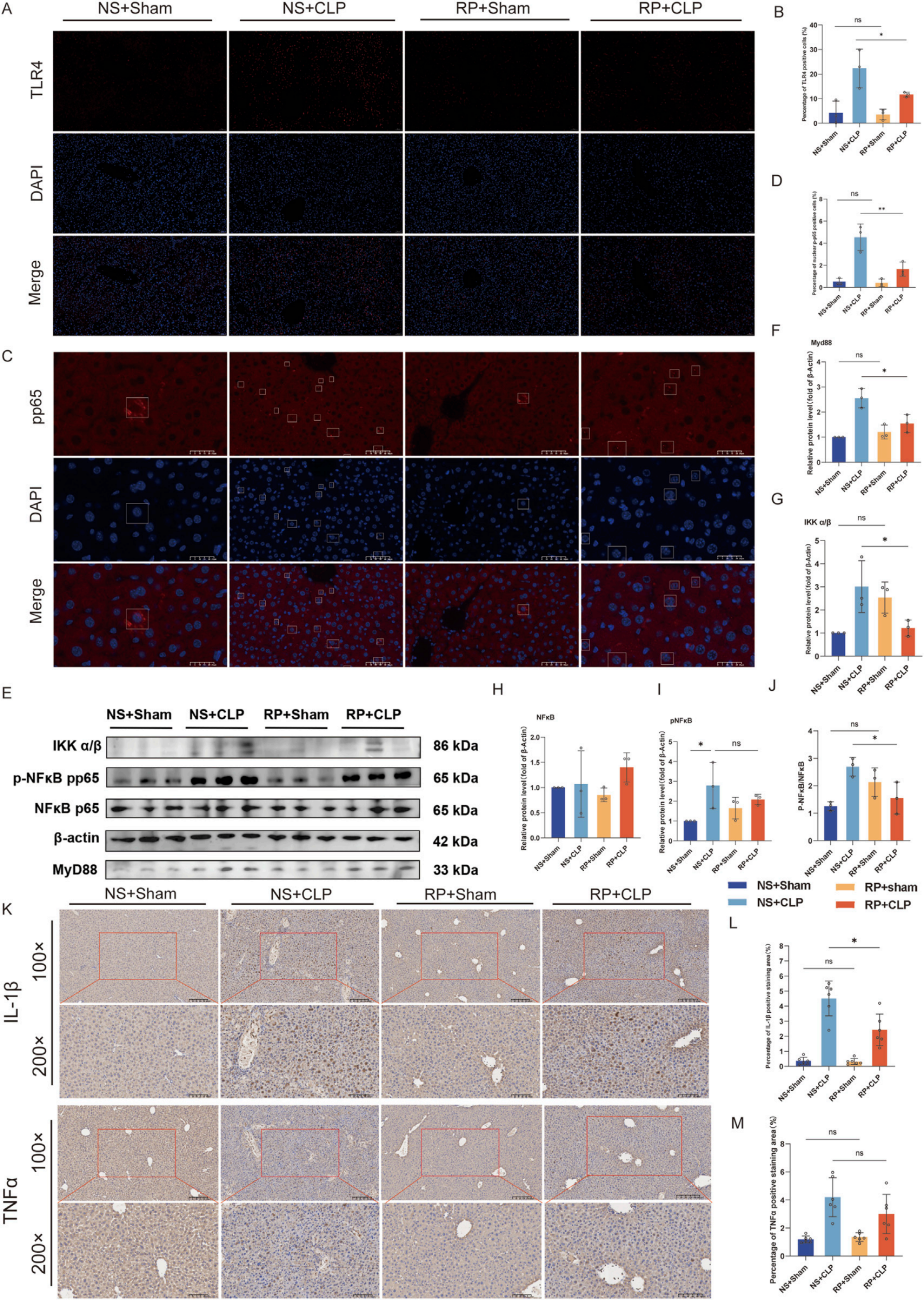
The beneficial effects of Radix Pseudostellariae polysaccharides (RPPS) on sepsis progression are associated with the TLR4/NF-κB pathway. **(A)** Immunofluorescent staining of Toll-like receptor 4 (TLR4) in liver tissues. (×200 magnification, n = 3). **(B)** Quantification of the percentage of TLR4-positive cells. **(C)** Immunofluorescent staining of p-p65 in liver tissues. **(D)** Quantification of the percentage of p-p65-positive cells. (Bar = 50 μm, n = 3). **(E)** Representative Western blot images of the NF-κB pathway. **(F–J)** Expression and quantification of MyD88, IKK α/β, NF-κB (p65), and pNF-κB (pp65) proteins using Western blot (n = 3). **(K)** Immunohistochemical staining for IL-1β and TNFα (×100 and ×200 magnification). **(L,M)** Percentage of IL-1β and TNFα positive area in liver tissue (n = 6). Data are expressed as the mean ± standard error of mean, and the significance of the differences between groups was assessed using one-way analysis of variance (Tukey’s test). *p < 0.05, **p < 0.05.

Immunofluorescence staining was employed to detect TLR4 expression and the nuclear translocation of p-p65 in liver tissue (Figures 7A,C). TLR4 expression on the cell membrane was lower in the RP + CLP group compared to that in the NS + CLP group (Figure 7B). Immunofluorescence staining revealed that, in comparison to the sham group, the nuclear translocation of p-p65 increased following CLP; however, this increase was significantly attenuated in the RP + CLP group relative to the NS + CLP group (Figure 7D). Assessment of the levels of Myd88, IKKα/β, NF-κB, and phosphorylated NF-κB in liver tissues using Western blot (Figures 7E–J) revealed a significant increase in Myd88, IKKα/β, and p-p65/p65 levels in the livers of mice in the NS + CLP and RP + CLP groups following CLP, but the increase was less marked in the RP + CLP group, indicating that pretreatment with RPPS reduced liver injury.

Immunohistochemical staining for IL-1β and TNFα revealed that RPPS reduced CLP-induced liver inflammation (Figures 7K–M). These results suggest that RPPS ameliorate SLI by inhibiting TLR4/NF-κB pathway activation and decreasing the generation of inflammatory mediators.

## 4 Discussion

We systematically investigated the hepatoprotective effects of RPPS using a murine model of CLP-induced sepsis. Our findings revealed that RPPS alleviate SLI through a multifaceted mechanism involving intestinal barrier repair, gut microbiota modulation, and suppression of the TLR4/NF-κB pathway. These results confirm the efficacy of the traditional use of RS as an anti-inflammatory and antioxidant and provide novel molecular insights into its therapeutic potential.

In sepsis, the liver commonly exhibits pronounced inflammatory responses and oxidative stress, resulting in inflammatory cell infiltration and the release of pro-inflammatory cytokines, such as TNF-α, IL-1β, and IL-6, exacerbating liver damage (Li et al., 2023). Pathological features of SLI include portal phlebitis, central lobule necrosis, hepatocyte apoptosis, and steatosis (Elias et al., 2023). In this study, the survival rate of septic mice was significantly lower than that of mice undergoing sham surgery, and the liver pathology was consistent with SLI. Notably, survival, MSS, serum bilirubin levels, and liver histopathology significantly differed between the NS + CLP and RP + CLP groups, indicating that RPPS ameliorate sepsis and SLI. These results lay a foundation for mechanism research.

The intestinal barrier plays a pivotal role in SLI pathogenesis, as gut dysbiosis and barrier disruption facilitate bacterial translocation and systemic endotoxin release (Sun et al., 2020). RPPS effectively restored intestinal integrity by upregulating occludin expression, a critical tight junction protein, reducing bacterial translocation and subsequent hepatic endotoxin exposure. This restoration of barrier function was complemented by RPPS-mediated remodeling of the gut microbiota. Metagenomic analysis demonstrated significant enrichment of beneficial taxa, such as *Lactobacillus*, *Muribaculum,* and *Ligilobacillus murinus*, which are essential for short-chain fatty acid (SCFA) production, and suppression of pathogenic genera, such as *Escherichia coli* and *Klebsiella* (Kim, 2021). SCFAs, particularly butyrate, enhance intestinal barrier function through G-protein-coupled receptor signaling and inhibit NF-κB activation, attenuating systemic inflammation (Menni and Valdes, 2019; Yao et al., 2022). This dual action of RPPS, direct barrier repair and indirect anti-inflammatory effects via SCFAs, establishes a synergistic “gut–liver axis” regulatory loop, offering a holistic therapeutic strategy.

RPPS exert their anti-inflammatory effects by targeting the TLR4/NF-κB pathway, a central mediator of SLI (Wang et al., 2024; Zamyatina and Heine, 2020). TLR4 activation by pathogen-associated molecular patterns triggers MyD88-dependent recruitment of the IKK complex, leading to IκBα degradation and NF-κB p65 nuclear translocation (Zhou et al., 2025). Our results confirmed that RPPS suppressed TLR4 membrane expression, inhibited MyD88 and IKKα/β phosphorylation, and blocked NF-κB p65 nuclear translocation, reducing the release of pro-inflammatory cytokines, such as TNF-α and IL-1β. This mechanism aligns with previous mechanistic findings on herbal polysaccharides, such as Gardenia polysaccharides, which similarly inhibit TLR4/NF-κB signaling (Fang et al., 2022).

Current therapies for SLI often focus on isolated pathways, limiting their effectiveness and safety (Wang et al., 2014). Unlike glucocorticoids, which carry risks of immunosuppression and metabolic side effects, RPPS offer a safer profile by leveraging natural polysaccharides to modulate host-microbe interactions. Herbal remedies for hepatitis, such as wogonin (a flavonoid from *Scutellaria baicalensis*) (Dai et al., 2021) and *Lycium barbarum* polysaccharides (Gao et al., 2021), primarily exhibit anti-inflammatory effects but lack direct impacts on the gut microbiota. In contrast, RPPS integrate microbiota modulation, barrier repair, and anti-inflammatory activity into a unified strategy.

This study had some limitations. First, the reliance on a murine model necessitates validation in higher species to ensure clinical relevance. Second, although RPPS promote SCFA-producing bacteria, direct evidence linking microbial metabolites to hepatic outcomes requires further metabolomic profiling (Gong et al., 2019; Liang et al., 2022). Third, the bioavailability and dose-response relationships of RPPS have not been thoroughly investigated. In future studies, we will employ *in vivo* experimental techniques, such as radiolabeling, to conduct a detailed investigation into the absorption, distribution, and metabolism of RPPS in mice. We will also design experiments with a broader range of doses to systematically evaluate the protective effects of RPPS against SLI.

This study integrated metagenomics, liver proteomics, and network pharmacology to elucidate the “multicomponent, multi-target” characteristics of TCM. Our multi-omics approach identified the TLR4/NF-κB pathway as a central mediator and underscored the critical role of gut microbiota in modulating SLI. The predicted interaction between RPPS and TLR4 was supported by molecular docking analysis. Future investigations could employ surface plasmon resonance to directly quantify the binding affinity between RPPS and TLR4, thereby bridging the gap between computational predictions and empirical validation.

## 5 Conclusion

This study positions RPPS as a promising TCM-derived candidate for SLI management, exemplifying how herbal polysaccharides can holistically target the gut–liver axis. By harmonizing traditional knowledge with modern systems biology, the findings of this study advance the scientific understanding of RP and facilitate the development of novel treatment options.

## Data Availability

The metagenomic data and proteomic data supporting the findings of this study has been deposited in the CNGB Sequence Archive (CNSA) with the accession numbers CNP0008017 and CNP0008016, respectively. Further inquiries can be directed to the corresponding authors.
