# Supplementary material for: Radix Pseudostellaria polysaccharides alleviate sepsis-induced liver injury by modulating the gut microbiota via the TLR4/NF-κB pathway
Source: Front Pharmacol. 2025 Sep 24;16:1658147. doi: 10.3389/fphar.2025.1658147 (PMC12504199; doi:10.3389/fphar.2025.1658147)
Supplement: Supplementary file 1 [file DataSheet1.doc]

**Supplementary Information**

**Radix Pseudostellariae polysaccharides alleviate sepsis-induced liver injury by modulating gut microbiota via the TLR4/NF-****κB pathway^^[[1]](#footnote-0)^^**

Zhuolin Wang^a,b,c,†^, Xiaohong Lin^a,b,†^, Jianfeng Wu^d,†^, Chanyuan Su^a^, Yukun Luo^a,c,*^, and Guangwei Yu^a,b,*^

^a^Department of Emergency, Fujian Medical University Union Hospital, Fuzhou, Fujian, China

^b^Fujian Key Laboratory of Vascular Aging, Fujian Medical University, China

^c^Department of Cardiology, Fujian Medical University Union Hospital, Fuzhou, Fujian Province, China

^d^Department of Neurosurgery, Fujian Medical University Union Hospital, Fuzhou, Fujian, China

†: These authors contributed equally.

*** Corresponding authors:**

Yukun Luo: Email: luoyukun@hotmail.com

Guangwei Yu: Email: ygwei1984@fjmu.edu.cn

# Materials and methods

# Extraction of RPPS

The RPPS were prepared via water extraction and ethanol precipitation, followed by solvent (acetone and ethanol) fractionation (Huang et al., 2022). RP roots were sourced from Fujian Mindong Rejuvenation Pharmaceutical Co., Ltd. (Ningde, China). The roots (300 g) were ground and extracted by decoction twice. The extract was concentrated under vacuum and centrifuged (3000 rpm, 30 min) for particulate matter removal. The supernatant was precipitated using 80% ethanol. The precipitate was dissolved in water and dialyzed against distilled water (cutoff value set at 3.5 kDa). The non-dialyzed fraction was freeze-dried to yield the polysaccharides (yield 25%) and the molecular weight is: 1.8×104 Da.

# Untargeted metabolomics by liquid chromatography-mass spectrometry

Liquid chromatography separation was performed using a Vanquish Horizon UHPLC system (Thermo Fisher Scientific, USA) equipped with an ACQUITY UPLC HSS T3 column (dimensions: 100×2.1 mm, particle size: 1.8 μm; sourced from Waters, Milford, MA, USA), maintained at a temperature of 40°C. The mobile phase, comprising aqueous 0.1% formic acid (solvent A) and acetonitrile (solvent B), was delivered at a steady flow rate of 0.4 mL/min. The acquired data were processed using Progenesis QI software.

**Pathological score of colon and liver**

A score from 0 to 4 was assigned considering the number of lesions and their severity in the colon, based on the number of goblet cells and crypt depth. The changes were graded as: grade 0 (no change from normal tissue); grade 1 (one or a few multifocal mononuclear cell infiltrates in the lamina propria accompanied by minimal epithelial hyperplasia and slight to no depletion of mucus from goblet cells or crypt); grade 2 (lesions involving more of the intestine or a greater number of lesions than those in grade 1); grade 3 (lesions involving a larger area of mucosa or a greater number of lesions than those in grade 2); grade 4 (lesions usually involved most of the intestinal section and more severe lesions than those in grade 3 (Shin et al., 2021). The pathological score of the liver was evaluated based on previous studies (Sun et al., 2020). Briefly, the parameters for inflammation, thrombus formation, and necrosis were graded on a scale of 0–4, with 0 defined as “absent” and 4 defined as “severe.” The total “histological score of liver” was expressed as the sum of the scores for each parameter. All scores were assessed separately by two independent pathologists.

## Results

## Qualitative analysis of RPPS

# The prepared RPPS (see Extraction of RPPS) was subjected to detection and analysis. The total ion current (TIC) diagram of the samples in positive and negative ion modes exhibited high resolution of the various indicators, with well-shaped peaks and relatively uniform distribution (Supplementary Fig. S1).


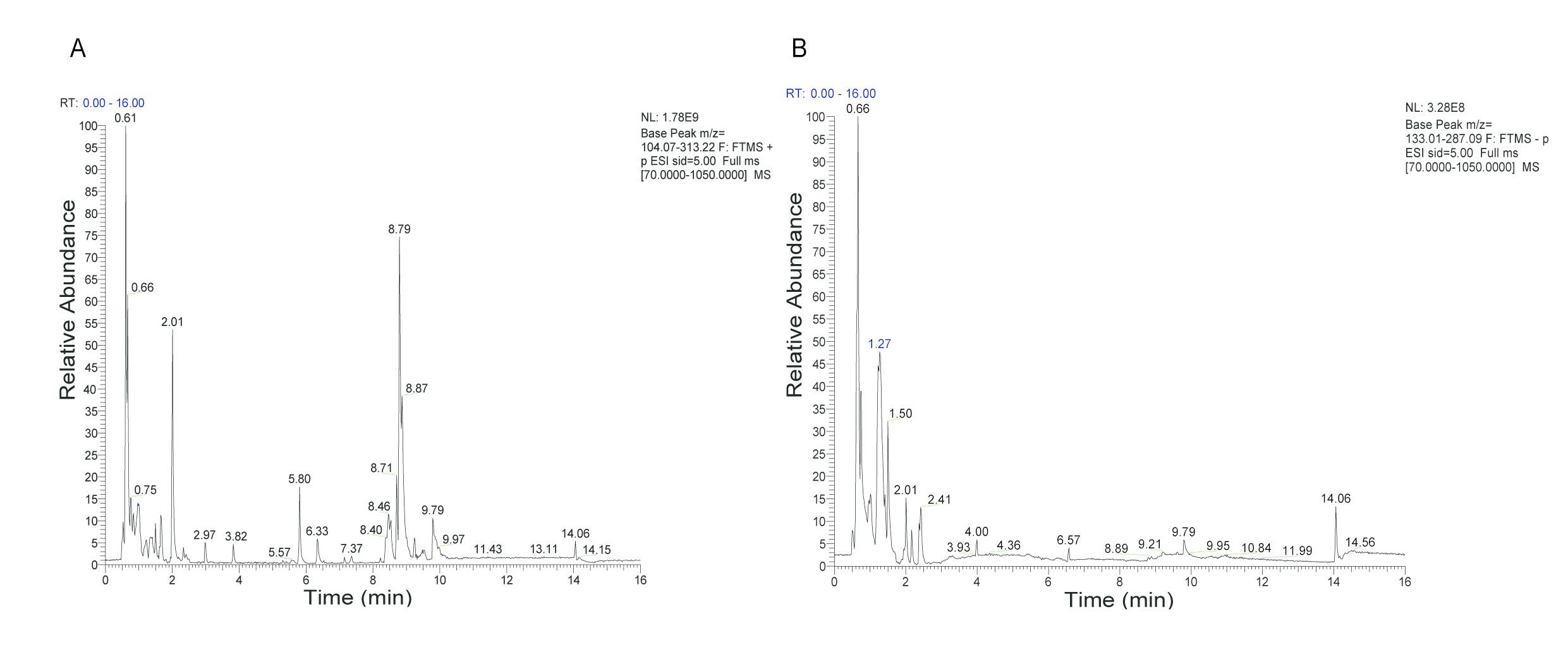


Supplementary Fig. S1. Total ion current (TIC) plots. (A) Positive ion mode. (B) Negative ion mode.

## The TCMSP database analysis and SWISSADME prediction analysis


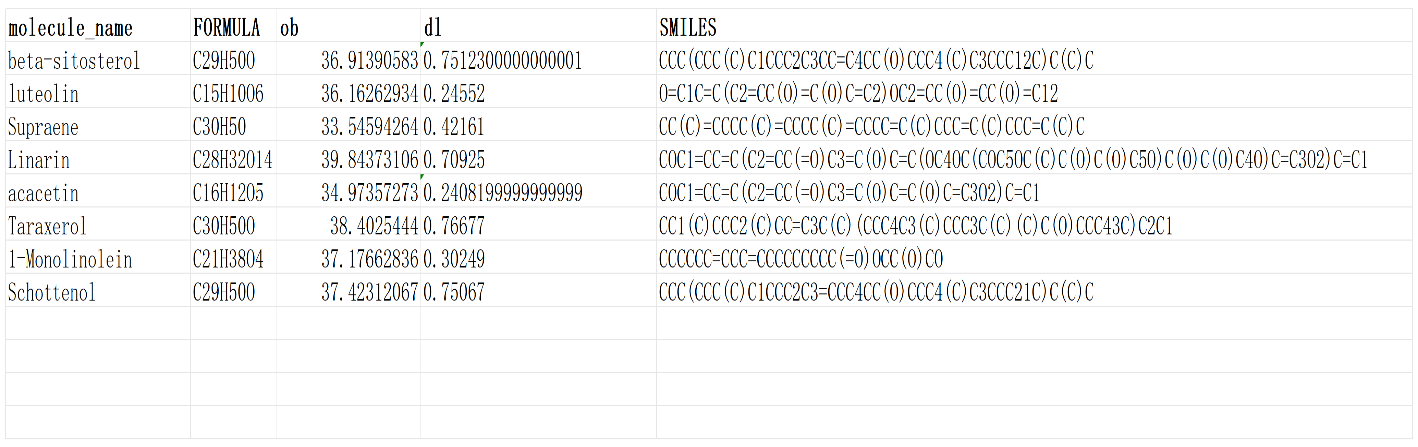


Supplementary Fig. S2 TCMSP_metabolites

Supplementary Fig. S3 SWISSADME_metabolites


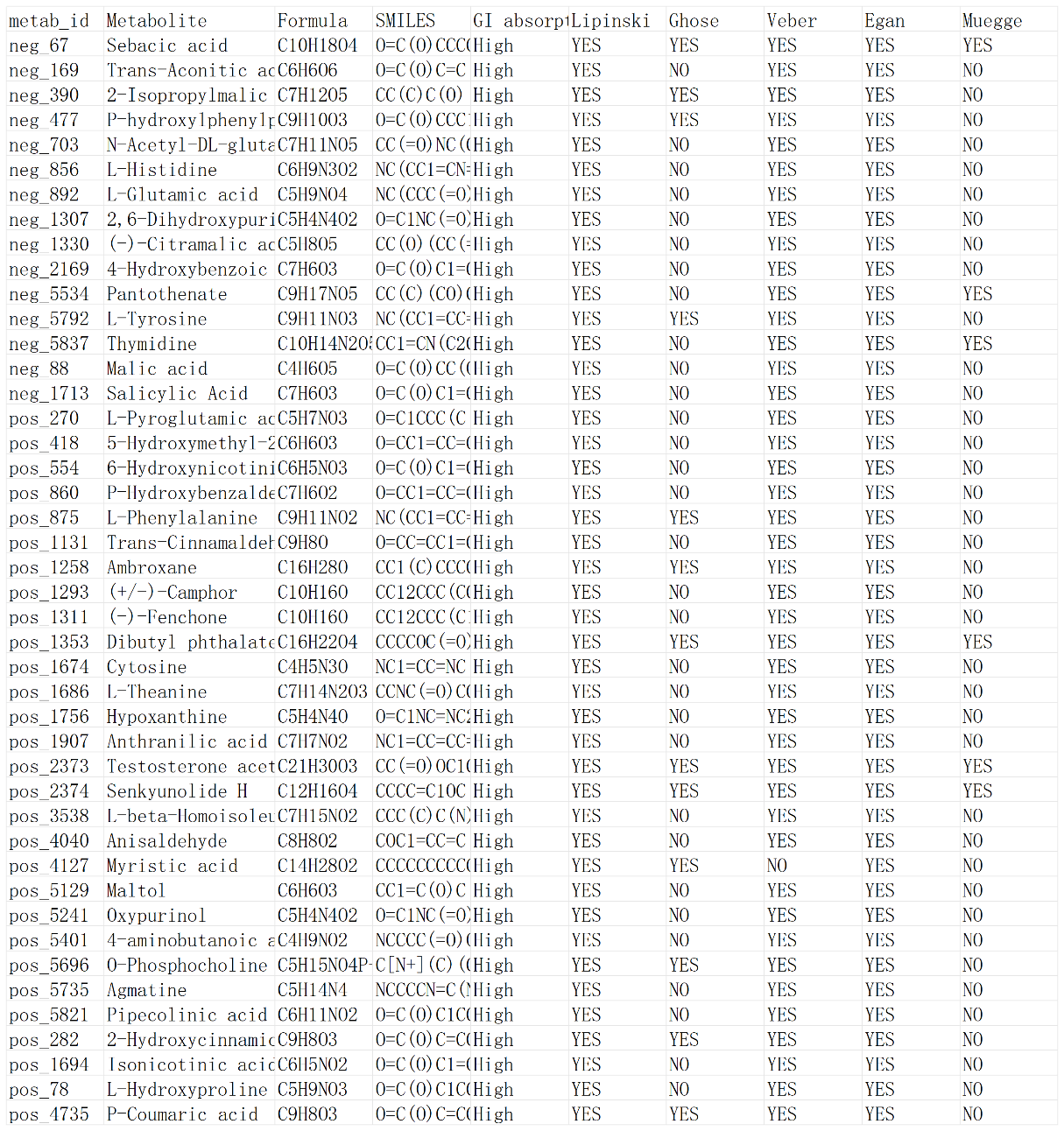

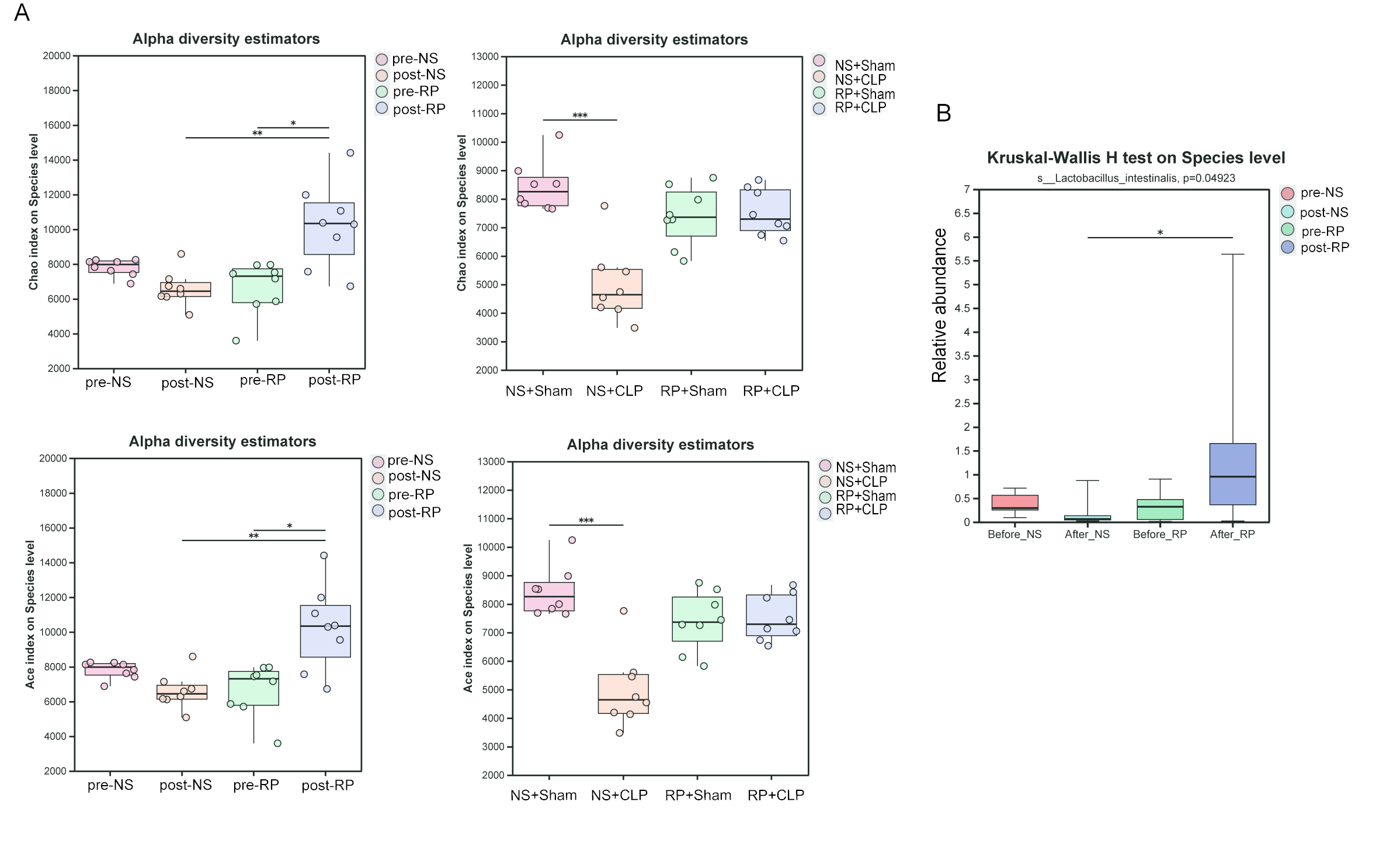


Supplementary Fig. S4. RPPS modulates the composition of gut microbiota. (A) The Chao and ACE index results. (B) The relative abundance of *Lactobacillus* across different groups.

**References**

Huang, J., Liu, D., Wang, Y., Liu, L., Li, J., Yuan, J., Jiang, Zhihong, Jiang, Zebo, Hsiao, W.W., Liu, H., Khan, I., Xie, Ying, Wu, J., Xie, Yajia, Zhang, Y., Fu, Y., Liao, J., Wang, W., Lai, H., Shi, A., Cai, J., Luo, L., Li, R., Yao, X., Fan, X., Wu, Q., Liu, Z., Yan, P., Lu, J., Yang, M., Wang, L., Cao, Y., Wei, H., Leung, E.L.-H., 2022. Ginseng polysaccharides alter the gut microbiota and kynurenine/tryptophan ratio, potentiating the antitumour effect of antiprogrammed cell death 1/programmed cell death ligand 1 (anti-PD-1/PD-L1) immunotherapy. Gut 71(4), 734–745. <https://doi.org/10.1136/gutjnl-2020-321031>

Shin, J., Noh, J.-R., Choe, D., Lee, N., Song, Y., Cho, S., Kang, E.-J., Go, M.-J., Ha, S.K., Chang, D.-H., Kim, J.-H., Kim, Y.-H., Kim, K.-S., Jung, H., Kim, M.H., Sung, B.-H., Lee, S.-G., Lee, D.-H., Kim, B.-C., Lee, C.-H., Cho, B.-K., 2021. Ageing and rejuvenation models reveal changes in key microbial communities associated with healthy ageing. Microbiome 9, 240. <https://doi.org/10.1186/s40168-021-01189-5>

Sun, J., Zhang, J., Wang, X., Ji, F., Ronco, C., Tian, J., Yin, Y., 2020. Gut-liver crosstalk in sepsis-induced liver injury. Crit. Care Lond. Engl. 24(1), 614. <https://doi.org/10.1186/s13054-020-03327-1>

1. [↑](#footnote-ref-0)
